# Orthostatic Hypotension and Mortality in Elderly Frail Patients: A Retrospective Cross-Sectional Study: Erratum

**DOI:** 10.1097/01.md.0000469902.81675.20

**Published:** 2015-07-17

In the article “Orthostatic Hypotension and Mortality in Elderly Frail Patients: A Retrospective Cross-Sectional Study”,^1^ which appeared in Volume 94, Issue 24 of *Medicine*, Dr. Yan Press's name was incorrectly spelled as Press Yan in the byline. The article has since been corrected online.
